# The Start of a Robotic Kidney Transplant Program: Institutional Step-by-Step Technique

**DOI:** 10.1590/S1677-5538.IBJU.2025.0532

**Published:** 2025-11-27

**Authors:** Alessandro Antonelli, Rostand Emmanuel Nguefouet Momo, Paola Donato, Gabriele Ugolini, Giovanni Corghi, Simone Priolo, Cristina Buttazzoni, Francesco Nacchia, Riccardo Bertolo

**Affiliations:** 1 University Hospital of Verona Urology Unit Department of Surgical Sciences Verona Italy Department of Surgical Sciences, Urology Unit, University Hospital of Verona, Verona, Italy; 2 University Hospital of Verona Kidney Transplant Center Department of Surgical Sciences Verona Italy Department of Surgical Sciences, Kidney Transplant Center, University Hospital of Verona, Verona, Italy; 3 University Hospital of Verona Intensive Care and Anesthesia B Unit Verona Italy Intensive Care and Anesthesia B Unit, University Hospital of Verona, Verona, Italy

## Abstract

**Purpose:**

To report our institutional technique for robot-assisted kidney transplantation (RAKT) (1, 2) in a detailed, step-by-step manner.

**Materials and methods:**

This is a case of RAKT from a living donor successfully performed at our institution. A 29-year-old male with end-stage renal disease secondary to focal segmental glomerulosclerosis, undergoing hemodialysis with a baseline serum creatinine of 1035 μmol/L at admission, received a left kidney donated by his 55-year-old mother. Preoperative evaluation confirmed one HLA mismatch (0-0-1) and ABO compatibility, making the patient suitable for living donation.

**Results:**

The procedure was performed using the da Vinci Xi robotic system (Intuitive, Sunnyvale, CA, USA). The recipient was placed in a 23° Trendelenburg position. Four robotic ports were aligned above the umbilicus, and two additional ports were used for the assistant. Graft introduction was performed via a 7-cm Pfannenstiel incision using an Alexis O Wound Protector-Retractor with Laparoscopic Cap (Applied Medical, Rancho Santa Margarita, CA, USA). Following robotic living donor nephrectomy, extracorporeal bench preparation was performed (warm ischemia time = 4 min; cold ischemia time = 239 min). RAKT was then completed with intracorporeal vasculares anastomoses using 5-0 Gore-Tex sutures (warm ischemia time = 45 min), and ureteral reimplantation according to the Lich-Gregoire technique, performed with 4/0 monofilament suture (3). The surgery was uneventful, with excellent graft reperfusion and no perioperative complications. Postoperative renal Doppler ultrasound and radionuclide renal scan were normal. Serum creatinine and eGFR at discharge were 1.45 mg/dL and 62 mL/min, respectively (4).

**Conclusions:**

Our experience confirms the feasibility and safety of RAKT with a living donor in a selected setting, supporting further integration of robotic assistance into renal transplantation programs.

**Available at:**VIDEO

## Data Availability

Uninformed
